# Dermatomyositis as a Paraneoplastic Syndrome Secondary to Carcinoma of Cervix: A Rare Clinical Association

**DOI:** 10.1002/ccr3.73605

**Published:** 2026-09-24

**Authors:** Manisha Chapagain, Paras Adhikari, Niranjan Pudasaini, Sabin Poudel, Bijay Timilsina

**Affiliations:** ^1^ Department of Dermatology Shree Birendra Hospital Kathmandu Nepal; ^2^ Nepalese Army Institute of Health Sciences Kathmandu Nepal

**Keywords:** autoimmune, cervical cancer, dermatomyositis, paraneoplastic syndrome, prednisolone

## Abstract

Dermatomyositis (DM) is a rare idiopathic inflammatory myopathy characterized by symmetric proximal muscle weakness and distinctive cutaneous manifestations. It is frequently associated with underlying malignancies, making recognition of paraneoplastic features crucial. We report the case of a 61‐year‐old postmenopausal woman who presented with progressive proximal muscle weakness, dysphagia, and characteristic skin lesions over the face, neck, and extremities for 6 months. Thorough evaluation with serology, electromyography, and muscle biopsy confirmed the diagnosis of DM. Further investigation revealed an underlying cervical mass, and biopsy established the diagnosis of non‐keratinizing squamous cell carcinoma of the cervix (Stage IIB). The patient showed partial improvement with systemic corticosteroids and demonstrated significant clinical recovery in both muscle strength and dysphagia following concurrent chemoradiotherapy. This case highlights the importance of evaluating patients with new‐onset DM for occult malignancy and underscores the rare but noteworthy association between DM and cervical carcinoma.

## Key Clinical Message

Dermatomyositis (DM) is an uncommon inflammatory myopathy that warrants thorough evaluation for an underlying malignancy, particularly when presenting in older adults. This case underscores the importance of recognizing DM as a potential paraneoplastic manifestation of cervical squamous cell carcinoma, where timely oncologic treatment can lead to significant improvement in neuromuscular and cutaneous symptoms.

**Abbreviations**
ANA: antinuclear antibody
CEA: carcinoembryonic antigen
CPK: creatinine phosphokinase
DM: dermatomyositis
LDH: lactate dehydrogenase
MRC: Medical Research Council

## 1 Introduction

Dermatomyositis (DM) is a rare idiopathic inflammatory myopathy characterized by progressive symmetric proximal muscle weakness and distinctive cutaneous manifestations, such as heliotrope rash, V sign, Shawl sign, and Gottron's papules. It is often associated with underlying malignancies, making it a well‐recognized paraneoplastic syndrome [1]. The association between DM and various malignancies has been well‐documented, though it is a rare finding; among them, the most commonly reported cases are cancers of the lung, breast, ovary, testis, and gastrointestinal tract [2]. An extensive review of the literature turned up a small number of additional cases of DM in conjunction with cervical squamous cell carcinoma [3, 4, 5, 6, 7]. However, DM as a paraneoplastic manifestation of cervical carcinoma is relatively uncommon and requires further exploration [8].

Paraneoplastic syndromes are uncommon disorders associated with malignancies that cannot be explained by direct extension of the primary tumor or metastasis but result from an altered immune response to the neoplasm [3]. Recent literature suggests that immunogenic tumor cells have antigens recognized by cytotoxic T cells, and during the course of the immune response, similar antigens, which are present on normal tissues, are attacked, leading to vague symptoms [9]. Cervical cancer remains a significant global health issue, with 660,000 new cases annually, while 94% of 350,000 total deaths occurred in low and middle‐income countries where screening and early detection programs are often inadequate [10, 11]. Risk factors include immunocompromised state due to HIV, autoimmune disease or medications, smoking, use of birth control pills, and human papillomavirus infection [12]. The paraneoplastic nature of DM in cervical cancer highlights the importance of recognizing this association, as it may serve as an early indicator of underlying malignancy [4]. Early diagnosis and treatment of both the neoplasm and the paraneoplastic syndrome are crucial for improving patient outcomes [8].

This case report presents a rare instance of DM as a paraneoplastic syndrome owing to carcinoma of the cervix. An extensive review of literature and discussion of the clinical presentation, along with management strategies, attempts to add to the expanding body of information on this uncommon association.

## 2 Case History/Examination

A 61‐year‐old post‐menopausal woman presented in March 2025 with complaints suggestive of progressive proximal muscle weakness, reddish skin rashes, and dysphagia for 6 months (onset September 2024). Muscle weakness started from the proximal upper limbs, noticed as difficulty in combing hair and reaching out to overhead objects, which gradually progressed over a period of 2 months, making it difficult to stand from a sitting position (Medical Research Council [MRC] muscle strength Grade 3/5). She developed itchy reddish flat to raised skin lesions predominantly over the face, neck, and extensor aspects of the upper and lower limbs over a period of 6 months. She also had foul‐smelling vaginal discharge for the same duration. She complained of progressive difficulty in swallowing both solid and liquid foods for 2 months.

**FIGURE 1:**
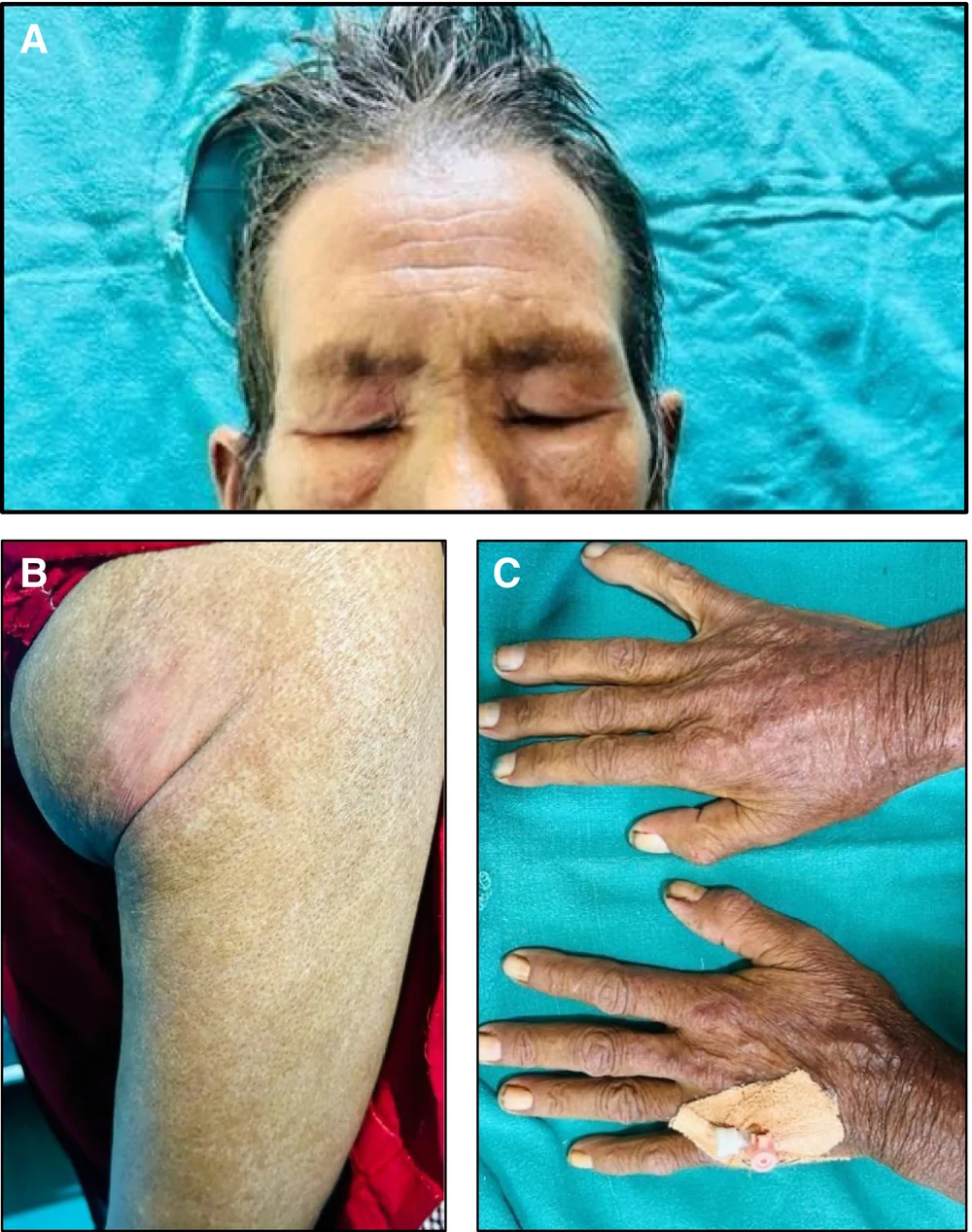
(A) Heliotrope sign. (B) Holster sign. (C) Gottron's papules.

Clinical examination revealed characteristic dermatological findings, including a heliotrope sign (Figure 1A), holster sign (Figure 1B), and Gottron's papules (Figure 1C).

## 3 Methods (Differential Diagnosis, Investigations, and Treatment)

**FIGURE 2:**
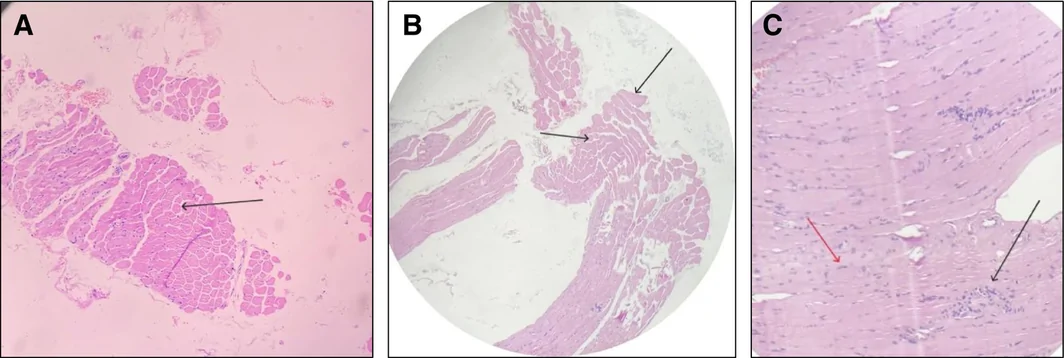
(A) Skeletal muscle fibers bounded by epimysium and muscle fasciculi enclosed by perimysium (black arrow) (4× magnification). (B) Mild variation in size of muscle fibers with decreased muscle diameter surrounded by clear spaces (black arrow) (10× magnification). (C) Mild perimysial inflammation (black arrow) along with occasional internalization of nuclei (red arrow) (40× magnification).

**FIGURE 3:**
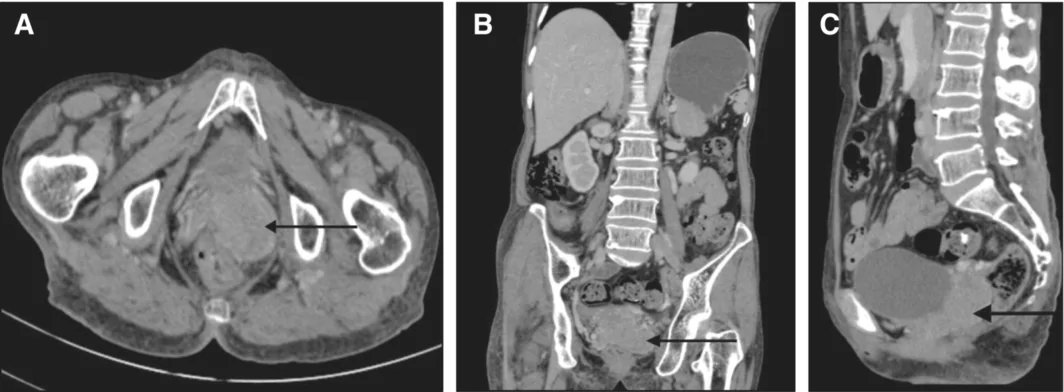
Contrast enhanced computed tomography scan images showing an irregular, ill‐defined, heterogeneous, enhancing soft tissue mass in the uterine cervix. (A) axial section; (B) coronal section and (C) sagittal section.

**FIGURE 4:**
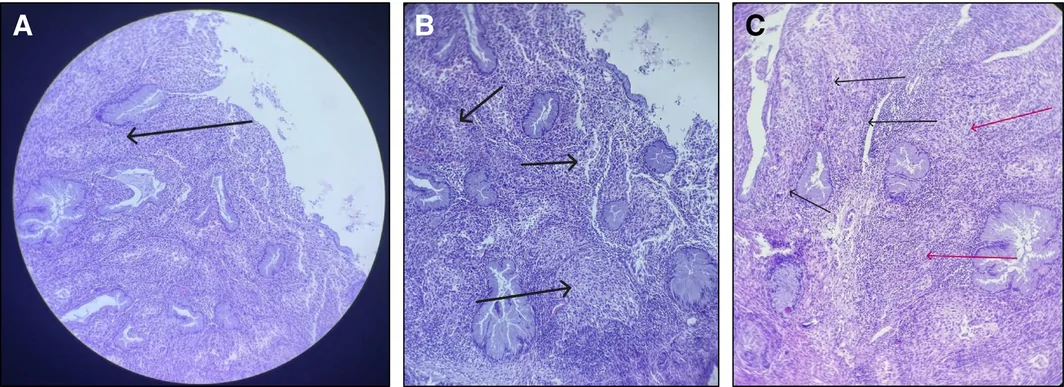
(A) Dysplastic stratified squamous epithelium (black arrow) on cervical biopsy (4× magnification). (B) Dysplastic stratified squamous epithelium with infiltration of tumor cells arranged in solid nests with no keratin pearl (black arrow) (10× magnification). (C) Non‐keratinizing squamous cell carcinoma with dysplastic epithelium (black arrow) and invasive foci (red arrow) (40× magnification).

Based on the aforementioned findings, DM, polymyositis, and systemic lupus erythematosus were made. On investigations, elevated LDH, creatine kinase, and electromyographic changes were observed, along with muscle biopsy findings and positive myositis‐specific antibodies (Table 1). Electromyography of the proximal upper and lower extremity muscles demonstrated typical myopathic features, including short duration, low amplitude, polyphasic motor unit action potentials with early recruitment and dense interference patterns, alongside localized fibrillations and positive sharp waves indicating active muscle fiber inflammation. Skeletal muscle biopsy (Figure 2A–C) taken from the right rectus femoris muscle revealed skeletal muscle architecture with muscle fibers bounded by epimysium and arranged in fascicles surrounded by perimysium (4×). There was mild variation in muscle fiber size (10×) magnification with evidence of perifascicular atrophy, showing reduced diameter muscle fibers at the fascicular border, some surrounded by clear spaces, along with focal perimysial inflammatory infiltrates and occasional internalization of nuclei (40×), with no evidence of malignancy suggestive of inflammatory changes. Autoimmune evaluation was further initiated with an antinuclear antibody (ANA) test performed by indirect immunofluorescence assay (IFA) on HEp‐2 cells using blood serum samples at a titration of 1:160, which showed an AC‐4 fine granular (nuclear speckled) pattern, suggestive of underlying autoimmune disease. For further evaluation, a commercially available Enzyme Linked Immunosorbent Assay (ELISA) immunopanel (MES‐100 Myositis Specific Autoantibody ELISA, Medical and Biological Laboratories Co. Ltd., Nagoya, Japan) was performed as per the manufacturer's instructions at a 1:100 dilution. The panel evaluated anti‐TIF1‐gamma, anti‐Mi‐2, anti‐MDA5, anti‐NXP2, and anti‐Jo‐1 antibodies. Positivity was defined according to the manufacturer's cutoff value (≥ 7.0 U/mL), which confirmed strong positivity for anti‐TIF1‐gamma antibodies, while all other panel antibodies were negative. On further investigation in March 2025, CEA level was found to be elevated, and contrast enhanced computed tomography (CECT) abdomen pelvis (Figure 3A–C) revealed an asymmetric mass of size 5.5 × 4.1 × 3.8 cm in the uterine cervix. The tumor was classified as Stage IIB as per the International Federation of Gynecology and Obstetrics, as the tumor extended beyond the parametrium. A heterogeneous enhancing left internal iliac lymph node measuring approximately 1.5 × 2.6 cm in size was noted, along with dilated and tortuous pelvic vessels predominantly on the left side. Subsequently, the lesion was visualized through colposcopic examination and biopsy of multiple friable grayish white tissue bits altogether measuring 3 × 1 × 0.5 cm was excised and later processed in hematoxylin and eosin staining (H&E) (Figure 4A–C), which showed the tissue was lined by dysplastic stratified squamous epithelium with infiltration of tumor cells exhibiting a moderate pleomorphism, eosinophilic cytoplasm, irregular nuclear border and hyperchromatic nuclei confirming the mass to be non‐keratinizing squamous cell carcinoma arising from high grade squamous intraepithelial lesion.

**TABLE 1:** Table showing relevant biochemical investigations.

Test	Values	Reference range
LDH	619 U/L	140–280 U/L
CEA	11.25 ng/mL	< 3 ng/mL (non smoker) < 5 ng/mL (smoker)
CPK	1049 U/L	30–135 U/L
Myositis‐specific antibodies	TIF1‐gamma positive	
ANA by IFA	Positive (1:160)	1:80

*Note:* These findings established the diagnosis of DM as a paraneoplastic syndrome secondary to carcinoma cervix.

Abbreviations: ANA, antinuclear antibody; CEA, carcinoembryonic antigen; CPK, creatinine phosphokinase; IFA, immunofluorescence assay; LDH, lactate dehydrogenase.

## 4 Conclusion and Result (Outcome and Follow‐Up)

She was initially managed with a high dose of Intravenous Methyl‐prednisolone (500 mg/day for 3 days) followed by oral prednisolone (1 mg/kg/day), leading to significant improvement in skin lesions but only mild improvement in muscle weakness (MRC Grade 3/5) and dysphagia. A multidisciplinary approach with a gynecologist and an oncologist was made for definitive management, and 2 weeks after her DM diagnosis, she was started on external beam radiotherapy (EBRT), 50.4 Gy in 28 fractions along with weekly cisplatin 40 mg/m^2^ per week. Significant improvement in dysphagia and muscle weakness (MRC grade improved to 4/5) was seen, and CPK decreased from 1049 to 600 U/L after 3 weeks of chemo‐radiotherapy. By 10 weeks post‐diagnosis (May 2025), CPK had decreased to 120 U/L, correlating with full recovery of motor power (MRC Grade 5/5) and a significant improvement in daily ambulatory activities was noted with no recurrence of DM symptoms. At this 10 week primary follow‐up endpoint, she was completing her final week of chemoradiotherapy with marked clinical regression of the cervical mass. She remains on a tapering dose of oral prednisolone and is undergoing ongoing regional oncological surveillance with 3 weekly follow‐up visits.

## 5 Discussion

DM is a rare autoimmune disease marked by symmetrical proximal muscle weakness and characteristic skin lesions such as Gottron papules, heliotrope rash, holster sign, Gottron sign, facial erythema, etc. While DM is a well‐known paraneoplastic syndrome, its association with cervical carcinoma is extremely rare. Females are more commonly affected than males. Its pathogenesis involves complement‐mediated vascular damage, particularly through the activation of complement factor C3 and deposition of the membrane attack complex [5].

Paraneoplastic DM is postulated to arise from an autoimmune response in which tumor antigens share epitopes with muscle and skin components, leading to immune cross‐reactivity [12]. Specifically, in HPV‐driven cervical carcinoma, viral oncoprotein‐induced somatic mutations in tumor TIF1‐gamma trigger anti‐TIF1‐gamma autoantibody production, which cross‐reacts with regenerating muscle and microvasculature. Large population‐based studies have demonstrated a significant association between DM and malignancy. In one cohort, approximately 32% of patients with DM developed cancer, with the highest risk observed around the time of DM diagnosis. The overall cancer risk in DM patients was three times higher than in the general population. While cancers like ovarian, lung, pancreatic, and stomach are classically linked to DM, the gynecological malignancies are usually ovarian while cervical remains notably an exception [2]. The dominant antigens found in cervical cancer (usually caused by human papilloma virus) are viral oncoproteins E6, E7 which are not normally expressed proteins due to which primary immune response is directed against these viral proteins and not on any other location [13, 14]. The symptoms show rapid improvement when the underlying malignancy is treated with radical chemoradiotherapy together with high‐dose steroids and intravenous immunoglobulin, and timely screening after treatment [5, 15].

In our patient, the temporal association between the onset of DM symptoms and the diagnosis of cervical cancer, along with the improvement in cutaneous and muscular symptoms following oncologic management after a duration of 2 weeks, supports a paraneoplastic etiology. The patient remains in outpatient follow‐up every 3 weeks to monitor clinical stability.

This case highlights the importance of recognizing DM as a potential paraneoplastic manifestation, particularly of cervical carcinoma, and emphasizes the role of a multidisciplinary approach in diagnosis and management.

## 6 Conclusion

To the best of our knowledge, this is a rare documented case of DM presenting as a paraneoplastic syndrome secondary to cervical squamous cell carcinoma. The patient showed partial improvement with corticosteroid therapy, with significant recovery in muscle strength and dysphagia following concurrent chemoradiotherapy. These findings underscore the importance of thorough malignancy screening in patients with new‐onset DM and highlight the role of coordinated multidisciplinary management combining immunosuppressives with definitive oncological management. It provides a clear basis for physicians to treat underlying occult neoplasms aggressively when paraneoplastic syndromes are suspected, as the mainstream of the treatment, especially in relevance to DM, which presents along with cervical carcinoma and promotes further research into shared molecular pathways between DM and cervical cancer to develop different modalities of targeted therapy.

## Author Contributions

**Manisha Chapagain:** conceptualization, writing – original draft, writing – review and editing. **Paras Adhikari:** conceptualization, writing – original draft, writing – review and editing. **Niranjan Pudasaini:** conceptualization, writing – original draft, writing – review and editing. **Sabin Poudel:** conceptualization, writing – original draft, writing – review and editing. **Bijay Timilsina:** conceptualization, writing – original draft, writing – review and editing.

## Funding

The authors have nothing to report.

## Consent

Authors have obtained informed written patient consent for use of their photographs and medical information to be published online and with the understanding that this information may be publicly available and discoverable via search engines. This consent form is not provided to the editorial office but has been retained by the author, and will be provided whenever requested.

## Conflicts of Interest

The authors declare no conflicts of interest.

## Data Availability

Data sharing not applicable to this article as no datasets were generated or analysed during the current study.
